# Machine learning for microfluidic design and control

**DOI:** 10.1039/d2lc00254j

**Published:** 2022-07-29

**Authors:** David McIntyre, Ali Lashkaripour, Polly Fordyce, Douglas Densmore

**Affiliations:** Biomedical Engineering Department, Boston University MA USA; Biological Design Center, Boston University Boston MA USA dougd@bu.edu; Department of Bioengineering, Stanford University Stanford CA USA; Department of Genetics, Stanford University Stanford CA USA; Chan-Zuckerberg Biohub San Francisco CA USA; Electrical & Computer Engineering Department, Boston University Boston MA USA

## Abstract

Microfluidics has developed into a mature field with applications across science and engineering, having particular commercial success in molecular diagnostics, next-generation sequencing, and bench-top analysis. Despite its ubiquity, the complexity of designing and controlling custom microfluidic devices present major barriers to adoption, requiring intuitive knowledge gained from years of experience. If these barriers were overcome, microfluidics could miniaturize biological and chemical research for non-experts through fully-automated platform development and operation. The intuition of microfluidic experts can be captured through machine learning, where complex statistical models are trained for pattern recognition and subsequently used for event prediction. Integration of machine learning with microfluidics could significantly expand its adoption and impact. Here, we present the current state of machine learning for the design and control of microfluidic devices, its possible applications, and current limitations.

## Abstract 2

In this review article, we surveyed the applications of machine learning in microfluidic design and microfluidic control.

## Graphical abstract

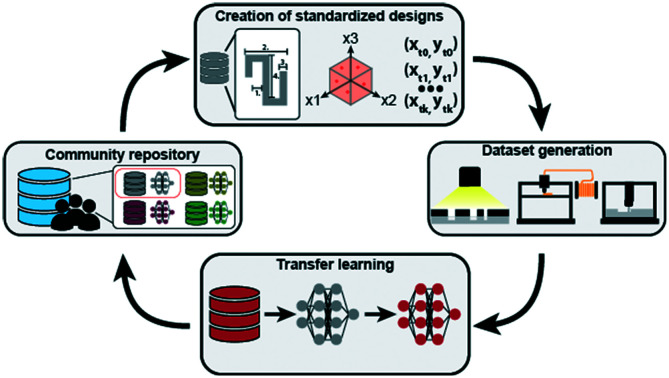

## 1 Introduction

Since its inception, microfluidics has been touted as a revolutionary platform to miniaturize biological and chemical experimentation. Microfluidics has been a core contributor to major technical progress in biotechnology, enabling the development and adoption of commercial platforms such as next-generation sequencing,^1^ single-cell RNA sequencing,^2^ or droplet digital PCR.^3^ Despite these successes, the impact of microfluidics is mostlylimited single-use cartridges inside of integrated bench-top devices, custom infrastructure within expert labs, or specific applications.^4^ As recently stated by Battat, Weitz, and Whitesides, wide adoption of microfluidic platforms has been limited by the complexity in the design, fabrication, and operation of custom devices that limit its reproducibility and generalizability.^5^ While fabrication can be outsourced (albeit at significant cost), microfluidic design and operation can require months to years of multiple “design-build-test” iterations to optimize performance.

**Fig. 1:**
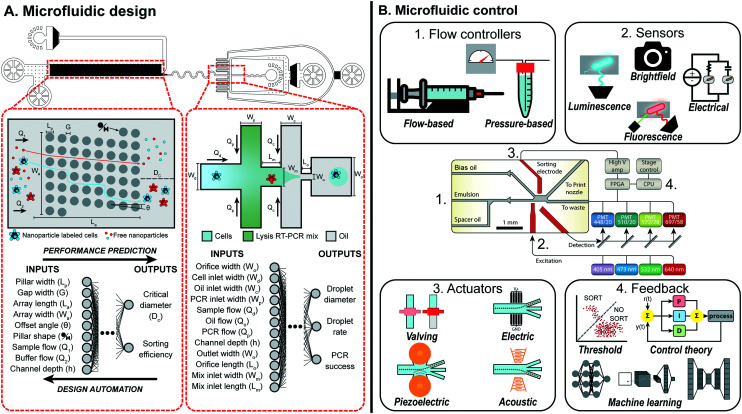
Overview of machine learning enabled automated microfluidic design and control. (A) Complex microfluidic devices, such as the MATE-seq platform,^9^ is comprised of two components, a deterministic-lateral-displacement array and droplet generator, which can be parameterized to describe both the physical design and experimental conditions. By mapping these parameters to a target performance, datasets can be generated and used to train machine learning models that predict the performance of each component. Design automation tools can use these models to automatically design each component such that the desired performance is achieved while adhering to design constraints. Figure reproduced from Ng *et al.*, 2019 (ref. 9) with permission from the Royal Society of Chemistry. (B) Microfluidic devices, such as a droplet sorter, can consist of a series of vital non-fluidic modules (schematic copyright 2017 National Academy of Sciences).^10^ These include: (1) flow controllers to drive behavior of the device; (2) sensors to measure and quantify occurring phenomena; (3) actuators to manipulate device behavior on the single-event level; and (4) feedback systems to respond to sensor information and intelligently control device behavior *via* the other modules. Machine learning provides a sophisticated feedback system to intelligently link modules together to perform complex tasks in real-time.

Machine learning (ML), the use of trainable statistical models to recognize patterns and predict future behavior, is a promising method to bridge the knowledge gap between experts and end-users and automate the design and operation of microfluidics. ML models range in complexity from a simple linear regression to deep neural networks (NNs). While deeper models can handle more complex datasets and make superhuman inference, they can be data, time, and cost intensive. Detailed reviews exist summarizing the different models to use for microfluidic^6^ and biological^7^ applications, as well as the hardware infrastructure needed to generate high-quality and complex datasets to train ML models.^8^ Elegant integration of machine learning in microfluidic design, testing, and optimization would eliminate many barriers to adoption in research and development, increasing the success rate and speeding up the commercialization of such platforms. In this review, we will survey current methods to simplify the design and operation of microfluidics with ML (Fig. 1).

### 1.1 Data in microfluidics

ML is most effective when there is (1) a quantifiable performance, (2) the ability to generate lots of data, and (3) a system that cannot be adequately modeled from first principles. The high-throughput and sensitive measurements up to the single-cell level as well as the complexity of devices makes microfluidics a good candidate to both generate data to train ML models and benefit from their predictive power. While model-based simulations are infinitely scalable, they can be computationally intensive and/or have simplifying assumptions that propagate error. Simpler fluidic components can be modeled *via* circuit analogies or numerical simulations. However, much of microfluidics is too complex for accurate modeling, for example in multi-phase flows, inertial processes, or when the performance metric is biological or chemical (synthesis yields, stochastic expression, morphology, *etc.*). ML is a good fit to predict such behaviors, yet requires diverse methods for data acquisition. Multiple sensing modalities are compatible with microfluidics depending on the phenomena to measure, each requiring different infrastructure and signal processing.

Most microfluidic operators have access to a camera to optically measure the fluid dynamics within their devices. This can range from a cell-phone to a custom, high frame-rate camera for high-throughput applications such as droplet microfluidics. Particularly high-throughput imaging of single cells in microfluidic devices (>10 000 cells per second) can be achieved through optofluidic time-stretch microscopy.^11^ Images and videos captured with cameras are simple to acquire and capture broad device performance yet can be data-intensive. Convolutional Neural Nets (CNNs) are ML models particularly suited for image datasets, yet can require a large amount of data and significant preprocessing to make accurate predictions.^12^ In many cases, inputting raw data into ML models is an unnecessarily inefficient and computationally expensive process. Design and operational conditions can be parameterized to describe the event; when combined with well-defined performance metrics (droplet generation rate,^13^ mixing index,^14^ inertial focusing,^15^*etc.*) all samples can be reduced to tabular datasets that enables quick model training with simpler architectures. Dataset labeling can be done manually if small enough or automated with image analysis and computer vision packages such as OpenCV.^16^

Measuring the occurrence of biological phenomena *via* a fluorescent or luminescent marker allows for researchers to quantify a specific event of interest. These modalities can be integrated into microfluidic processes, whether through embedding excitation sources and sensors into devices or manipulating a sample upstream of a flow cytometer or fluorescence-activated cell sorting (FACS) machine.^17,18^ In embedded devices with a detector such as a photomultiplier tube (PMT), fluorescence or luminescence levels are outputted as a voltage signal proportional to the light measurement.^19^ Optical filtering can be applied to these systems to multiplex measurements to other fluorophores. This data modality is compatible with a variety of ML models, from simple classification to a complex CNN when combined with optical imaging.^20^

One major limitation to fluorescent and luminescent sensing is the need for an engineered label, which can be difficult to integrate or compete with the target pathway. Additionally, the bandwidths of common fluorescent labels restricts the number of markers measured simultaneously. Label-free measurements are a good alternative to these systems when the information needed or engineering challenge for label insertion are not suitable for fluorescence or luminescence. Light detectors can be re-purposed to measure the absorbance of a sample, providing information on the cell growth within the microfluidic device.^21^ Electrical sensing (impedance, capacitance, voltage, *etc.*) can also be used to detect droplets or cells as they pass through a microfluidic device or bind to functionalized electrodes within channels, detecting different sample position and velocity, cell types or state, or cell-growth over time.^22^ Recently, advanced label-free techniques have been integrated into microfluidics, namely Raman spectroscopy^23–25^ and mass spectroscopy.^26,27^ Full and simple integration of such methods would provide sophisticated label-free measurement of biological and chemical samples at scale, yet many technical challenges exist in standardizing workflows and increasing the throughput of such methods to be accessible to the broader community and used to generate datasets for machine learning.

## 2 Performance prediction and design automation of microfluidic systems

Microfluidic device design is commonly guided by analytical, empirical, and numerical models.^28^ Analytical models are typically limited to relatively simple microfluidic operations;^29,30^ scaling laws, explicit mathematical equations that are fit to predict physical phenomena, can capture the general dynamics of complex phenomena, yet, simplifying assumptions limit their generalizability.^31^ Numerical models are suitable for rapid *in silico* experimentation, but, they can be error-prone and require mindful data parsing, specifically in complex flow fields.^30,32^ Even with these guides, microfluidic design is generally an expensive and iterative process, especially so for multi-component microfluidic devices,^9^ photo-lithographic fabrication methods,^33^ or when optimizing for poorly characterized biological samples.^34,35^

Adoption of ML has already shown promise to reshape the microfluidic design process.^6^ However, the majority of ML models have been implemented to automate data analysis, not microfluidic design.^28^ In this section, we survey existing approaches for both performance prediction and design automation of microfluidic devices with an emphasis on the emerging ML models.

### 2.1 Performance prediction in microfluidics

#### 2.1.1 Non-machine learning based approaches

Laminar fluid flow in microchannels is one of the simplest microfluidic operations and can be modeled by the Hagen–Poiseuille law. Flow rates or pressure drops can be calculated analytically from the Hagen–Poiseuille equation, through simplifying assumptions that model behavior as analogous to a voltage drop or current in electrical circuits. Hagen–Poiseuille flow can be extended to most channel geometries, multiphase flows,^31,36,37^ and be used for designing branches to minimize hydraulic resistance and pressure drop.^38,39^ The hydraulic–electric analogy allows for modeling of sophisticated behavior, including microfluidic pneumatic circuits capable of digital computation with on-chip valves.^40–42^ Designing microfluidic networks with the hydraulic–electric analogy is thoroughly reviewed by Oh *et al.*^29^ In addition to laminar flow, analytical models with simplifying assumptions have been proposed for convective-diffusive transport in micromixers,^43–45^ inertial flows,^46–48^ acousto-microfluidics,^49^ magnetic separation,^50,51^ suspended microfluidic systems,^52^ and capillary flows.^53,54^

Since the Navier–Stokes equation does not have a generalizable solution, empirically or analytically-derived scaling laws are often used to approximate the fluid dynamics in microfluidics,^30^ including for micro-mixing,^55^ droplet generation and break up,^56–58^ inertial microfluidics,^59,60^ capillary flows,^54^ and acousto-microfluidics.^61,62^ Although scaling laws are powerful tools for describing complex microfluidic behavior, simplifying assumptions and bounded parameter spaces prevent their generalized use in accurate performance prediction for all flow regimes, fluid types, or microchannel geometries.^13,30,31^

Computational modeling of fluid dynamics can enable rapid, yet error-prone analysis of microfluidic performance,^63^ and has been used for multiphase flows,^64^ inertial microfluidics,^65^ acousto-microfluidics,^66^ capillary microfluidics,^67^ magnetophoresis,^68^ and microfluidic fuel cells.^69^ These numerical models can be combined with other methods to improve prediction accuracy. For example, numerical simulations were used in conjunction with the hydraulic–electric analogy to predict particle trajectory in a grid^70^ or solute concentration at the outlets of a randomly designed grid micromixer.^71^ In Wang *et al.*, a combination of COMSOL numerical modeling for solving the flow velocity field and a custom algorithm for modeling trajectories of 2D rigid particles, called MOPSA, was used to predict trajectories of cells, droplets, and other particles in microchannels, including deterministic lateral displacement devices.^72^ As computational power scales, such combined approaches will likely be used more broadly to adapt numerical models to new microfluidic phenomena. Furthermore, the ever-expanding availability of experimental data will better verify and tune numerical models, enabling its rapid expansion to new fluid and flow properties, geometries, and applications.

#### 2.1.2 Machine-learning based approaches

**Fig. 2:**
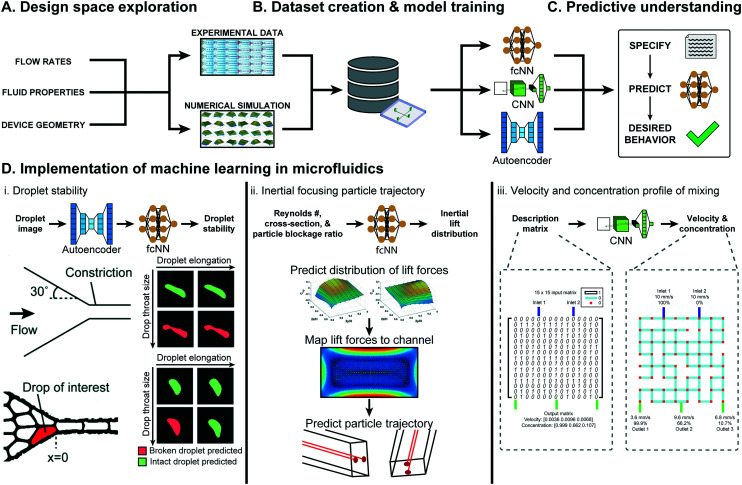
Overview of ML approaches in microfluidic performance prediction. (A) The performance of a microfluidic device is affected by the flow rates, fluid properties, device geometry, and material. (B) With informed sampling methods, this large design space can be explored experimentally or numerically to create a sufficiently sized dataset for training ML models. (C) Several classes of ML models, such as fully-connected neural networks (fcNN), convolutional neural networks (CNN), and autoencoders can be trained to gain generalizable predictive understanding of microfluidics. (D) Examples of ML-assisted performance prediction in several types of microfluidic devices. (D.i) An autoencoder in conjunction with a fcNN was used to predict droplet stability in tightly packed emulsions passing through a constriction. Figure reproduced from Khor *et al.*, 2019 (ref. 77) with permission from the Royal Society of Chemistry. (D.ii) ML models were used to predict the distribution of lift in broad range of operating conditions, which was then used to estimate particle inertial focusing in multiple cross-sections. Figure reproduced from Su *et al.*, 2021 (ref. 15) with permission from the Royal Society of Chemistry. (D.iii) CNNs were used to predict the solute concentrations and flow rates at the three outlets of a 2-inlet randomly designed grid micromixer. Figure reproduced from Wang *et al.*, 2021 (ref. 78) with permission from the Royal Society of Chemistry.

While ML has primarily been applied for automated data analysis,^6,73^ preliminary work has shown its potential to predict device performance and automate design. The high-throughput nature of microfluidics in tandem with automated data labeling allows for the creation of large experimental datasets from a single device.^20^ The recent introduction of rapid prototyping methods and computational models further accelerated the data generation process and expanded the number of unique devices that can be feasibly tested.^74–76^ ML models trained on large-scale datasets enable predictive understanding even in complex fluidic phenomena and high-dimensional design spaces, tackling one of the grand challenges in the field (Fig. 2A–C).

Droplet microfluidics is an application well-suited for ML-based performance prediction, as the complex fluid dynamics of multiphase flows prevent generalizable understanding.^31,79^ In Khor *et al.*, ML models predicted droplet stability within tightly packed emulsions passing through a constriction.^77^ The developed model, an 8-dimensional convolutional autoencoder for feature extraction and a two-layer fully connected classifier, was trained on 500 000 droplets and could predict droplet stability with 91.7% accuracy, in contrast to the 60% accuracy of conventional scalar descriptors (Fig. 2D.i). In Hadikhani *et al.*, ML models predicted the flow rate and concentration of isopropanol (IPA) used to generate water–IPA droplets.^80^ With a dataset of 6000 images of variable flow rates and 3600 images of variable IPA concentration, the developed models could predict IPA–water flow rate and concentration in a test-set, with 5.7% and 9.3% mean absolute percentage error (MAPE), respectively.

ML models have also gained momentum for performance prediction in microfluidic droplet generation. Mahdi *et al.* used ML to predict the size of water droplets in glycerin oil generated from a T-junction geometry.^81^ With 742 data points, the trained model took Reynolds and capillary numbers for both phases as inputs to predict droplet size with high accuracy (*R*^2^ ≈ 1) for multiple flow rates and fluidic properties within a single geometry. In Lashkaripour *et al.*, NNs predicted the droplet size, generation rate, and regime of flow-focusing droplet generation as a function of design geometry and flow conditions.^13^ Capillary number, flow rate ratio, and six geometric parameters were varied across 888 datapoints and used to train NNs that accurately predicted the generation regime (95.1% accuracy), droplet size (mean absolute error less than 10 μm), and generation rate (mean absolute error less than 20 Hz) for droplets with sizes and rates between 25–250 μm and 5–500 Hz, respectively. These ML models could also be extended to new aqueous solutions or oils through transfer learning with small-scale datasets. In Damiati *et al.*, ML models predicted poly(d,l-lactide-*co*-glycolide) (PLGA) microparticle size generated using flow focusing droplet generators and dichloromethane solvent evaporation.^82^ Data acquired over 223 different combinations of flow rates, PLGA concentrations, device types, and whether droplet or particle size is being predicted were used to train a model capable of predicting PLGA particle size (*R*^2^ greater than 0.94).

ML based performance prediction is also rapidly gaining traction in other areas of microfluidics. In Su *et al.*, ML models predicting microfluidic inertial lift distribution were trained on 14 160 simulated data points varying Reynolds number, channel cross-section shape, and particle blockage ratio.^15^ The predicted lift distribution is then mapped to a cross-section to predict particle migration with a Lagrangian tracking scheme, combining ML and numerical approaches (see Fig. 2D.ii). In Wang *et al.*, CNNs were used to predict the fluid velocity and solute concentration in randomly designed grid micro-mixers with two inlets and three outlets.^78^ The developed models were trained on a previously created simulated dataset of 10 513 randomly generated micro-mixer designs that varied the grid design, described by a 15 × 15 binary matrix, keeping inlet solute concentration and velocity constant (see Fig. 2D.iii).^83^ These models predicted outlet flow rates with an accuracy rate of 86.7% (assuming a threshold absolute error of 1 mm s^−1^) and could predict the outlet solute concentration of at least 94.5% of data-points in the test set with less than 30% absolute error.

### 2.2 Design automation of microfluidic systems

As surveyed above, the need for microfluidic platforms that are able to be readily re-purposed across applications can be met by ML models that can accurately map design and operation parameters to performance.^28^ Integration of these models with computer-aided design (CAD) frameworks would further enable microfluidic design automation, where a desired performance is translated to a device design and operating conditions.

Non-machine learning and machine learning based approaches, such as query-based methods or hydraulic–electric analogies, have been used for microfluidic design automation. Wang *et al.* demonstrated the first successful implementation of microfluidic design automation, collecting data from 10 513 numerically simulated, randomly design 8 × 8 grid micromixers and using these data to create a web CAD tool capable of designing devices that can produce a desired outlet solute concentration with less than 4% MAPE.^83^ In Grimmer *et al.*, the hydraulic–electric analogy was used to automate design of meander microchannels that deliver the desired resistance blocks and mixing ratios on-chip.^85^ The developed tool, Meander Designer, delivered a desired resistance block of 10 to 50 mbar min μL^−1^ as well as two meander blocks with different resistances and an integrated pressure pump used to mix at different ratios.

**Fig. 3:**
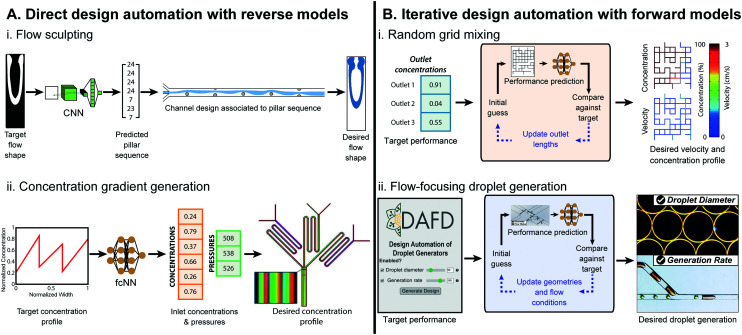
Examples of ML-assisted workflows for design automation of microfluidic devices. (A) Direct approaches use ML reverse models that convert the desired performance to microfluidic design parameters. (A.i) ML-assisted direct design automation were demonstrated for flow sculpting in inertial flows, figure reproduced from Stoecklein *et al.*, 2017 (ref. 84) licensed by CC BY 4.0; and (A.ii) for generating user-specified concentration gradients, figure redrawn from Hong *et al.*, 2020 (ref. 76) licensed by CC BY 4.0. (B) Iterative design automation uses ML forward models that convert microfluidic design parameters to the predicted performance in conjunction with a iterative search algorithm to convert the user specified desired performance to the necessary design parameters. Iterative design automation were demonstrated for (B.i) design automation of output solute concentrations and flow rates in randomly designed 2-inlet, 3-outlet grid micromixers,^71^ random mixer figure reproduced from Wang *et al.*, 2016 (ref. 83) with permission from the Royal Society of Chemistry; and (B.ii) for droplet diameter and generation rate in flow-focusing microfluidic droplet generators, figure reproduced from Lashkaripour *et al.*, 2021 (ref. 13) licensed by CC BY 4.0.

^84^ Reverse model CNNs were trained on 150 000 images and tested against 10 000 images generated *via* uFlow,^86^ an experimentally validated computational fluid dynamics (CFD) model, and could output pillar array designs that produced flow shapes with a pixel match rate (PMR) of 0.8 against the test set (a PMR of 0 denotes no image similarity, while a PMR of 1 is perfect) (Fig. 3A.i). Hong *et al.*, used ML models for design automation of concentration gradient generators.^76^ Here, 9-million data points were generated from an experimentally verified, physics-based model^87^ to train a fully-connected cascade NN that maps a desired concentration profile to inlet concentrations and pressures, delivering a specified concentration profile with a MAPE of 8.5% (Fig. 3A.ii).

Machine learning methods for microfluidic design automation often follow two possible approaches: direct design automation, where reverse predictive models directly convert performance metrics to design parameters, and iterative design automation, where forward models mapping design to performance are used in conjunction with iterative automated search algorithms (Fig. 3). Stoecklein *et al.* demonstrated one of the first uses of ML in microfluidic design automation, to sculpt flow using passive pillars in inertial fluid flow (1 < Re < 100).

In Ji *et al.*, ML was used for iterative design automation of randomly designed grid micromixers.^71^ The NNs were trained on 4320 simulated chips^83^ and mapped the length of output channels to output concentration. This could produce designs with outlet concentrations within 0.01 mol m^−3^ of the desired values for 91.5% of benchmarks, compared to its simulated performance (Fig. 3B.i). Lashkaripour *et al.* developed an ML-based iterative design automation tool, DAFD, for flow-focusing droplet generators using DI water and mineral oil.^13^ Forward models trained on 888 experimental data-points across 43 devices and 65 flow conditions could accurately predict droplet size, generation rate, and generation regime from input design geometry and flow conditions. These models were used in conjunction with an iterative search algorithm to enable design automation of flow-focusing droplet generators with less than 4.2% and 11.5% MAPE for droplet diameter and generation rate, respectively (Fig. 3B.ii).

Both direct and iterative approaches are proven methods for microfluidic design automation. Direct approaches face the challenge of many-to-one conversion (multiple designs could deliver the desired performance), that can be solved through either divide-and-conquer approaches with cascade NNs^76^ or mindful sampling of the design space to ensure a uniform training set distribution.^84^ Iterative approaches can also propose several designs that deliver the same performance. Nonetheless, by starting the optimization algorithm from multiple initial conditions and using a ranking scheme an optimal design can be achieved.^71^ Furthermore, the iterative design iteration approach enables design constraints (*i.e.*, fixed design parameters) to be specified by the user to ensure that the final design conforms to one or more user constraints in addition to delivering the desired performance.^13^

## 3 Microfluidic control

In practice, the operation of a microfluidic device can require as much expertise as its design and fabrication. While design automation can bring users extremely close to a desired performance, in particularly sensitive situations adaptive control of devices is needed to bring performance to and keep at a set point over the course of an experiment. Many applications of microfluidics require real-time feedback from sensor readouts to trigger pneumatic, acoustic, or electrical actuation, or a change in pump input.^88^ Furthermore, errors from fabrication or during operation can require a change in control parameters to achieve target behavior. Performance changes of varying magnitude can arise from errors in fabrication, variation in surface treatment (*e.g.* to tune hydrophobicity or hydrophilicity), surface fouling, clogging of channels with dirt or introduction of air bubbles, or inconsistencies in flow control. When being fed data from microfluidic sensors, ML can learn the behavior of a device on the fly and control active components in response to event detection.

### 3.1 Non-machine learning based methods

One early use of automated microfluidic control was in high-complexity valving networks, in which system states were predefined and programmed to perform complex tasks.^89^ In electrowetting-on-dielectric (EWOD) digital microfluidic systems, algorithmic feedback has been implemented to standardize upstream droplet generation and pathfinding on large-scale devices.^90,91^ For on-the-fly adaptation such as error correction from channel blockage, flexible microfluidics have been develop that change channel dimensions in response to a bias voltage change, clearing clogs in the system.^92^ Optimization of microfluidic functions have also been implemented with proportional-integral-derivative (PID) control and design of experiments (DoE) methods. PID control, an engineering tool commonly used to fix system behavior at a previously-identified set point, was applied to control the electric field voltage and frequency needed to keep particle density constant in microfluidic colloidal self-assembly.^93^ DoE has also been used to identify optimal conditions for liposome production.^94^ Integration with ML can significantly increase the breadth and complexity of intelligent microfluidic operation, streamlining the operation of expert devices.

### 3.2 Optimization of device performance

Two ML approaches, reinforcement learning (RL) and Bayesian optimization (BO), are particularly powerful tools to optimize behavior of a microfluidic device.^96^ In RL, an agent attempts to maximize a reward function based upon performance within an environment, whereas in BO the maximum of a black-box function is identified by building a surrogate model and iteratively testing different parameter combinations of that model.

**Fig. 4:**
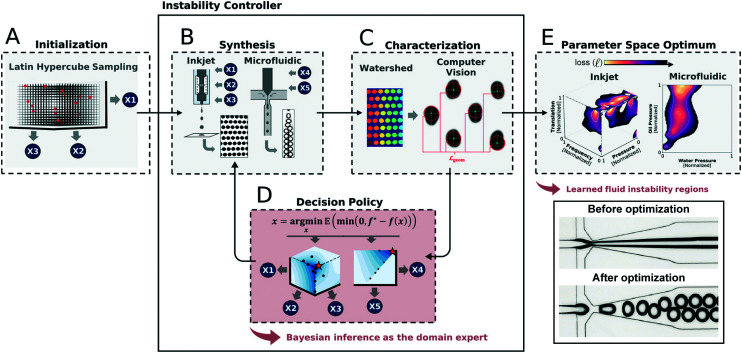
Example of an application of machine-guided microfluidic control in its implementation to optimize droplet generation at multiple length scales.^95^ (A) After an initial sampling of the parameter space, (B) a small-scale dataset is generated and (C) automatically analyzed using computer vision methods. (D) These results are then fed into a Bayesian decision policy that determines the next set of data to generate. (E) This iterative loop continues until performance is optimized and the boundaries of the stable droplet generation regime is identified. Reprinted with permission from Siemenn *et al.*, 2022.^95^ Copyright 2022 American Chemical Society.

Optimization of device performance is particularly attractive in droplet microfluidics, where having device output specifically match a user-defined droplet size or rate is critical. In Siemenn *et al.*, BO was used in tandem with computer vision to automatically identify regions of stable droplet formation and converge upon a user-defined droplet generation rate and size (Fig. 4).^95^ This process removes the need for domain knowledge and iterative experimental cycles; after initializing the model with 20 pseudo-random datapoints, the ML loop can converge upon a user-defined performance in 60 points total (20 initial and 4 batches of 10 algorithmically-requested points). In Dressler *et al.*, two RL algorithms, Deep-Q Networks (DQNs) and model-free episodic controllers (MFECs) were compared against human performance and each other in the ability to control both laminar flow between two different fluids as well as droplet generation between two immiscible fluids (water in oil emulsions).^97^ In each case, both models either matched or exceeded superhuman performance. Both systems can be iterated across different oil types and droplet composition and used to optimize more complex systems (*e.g.* double emulsion generation or liposome formation). In EWOD microfluidic devices, RL was used to adapt droplet routing across electrodes as performance decreases across the lifetime of the device.^98^

In continuous flow microfluidics, valves provide critical control of a device's operating state. Microfluidic valving can be exceedingly complex, in some cases requiring 1000s of valves.^99,100^ Abe, Oh-Hara, and Ukita built a proof-of-concept system for applying RL to the control of microvalving to set the flow rate of a persitstaltic pump.^101^ A 3-valve state was modeled as a Markov process, and was used to simulate flow rates produced from different state cycles. While used for a simple system, this principle could be particularly impactful when applied to more complex microfluidic valving networks.

### 3.3 Analysis and feedback of sensor output

One of the major benefits of microfluidics is its ability to measure phenomena with single-cell resolution. In sensing modalities with simple measurement data structures (fluorescence, luminescence, *etc.*), sample output level can be analyzed through a straight-forward pipeline such as a peak detection algorithm. In these cases, downstream device action can be determined by comparing peak heights against preset threshold values. In more complex signal responses (electrical, optical, *etc.*), sophisticated analysis such as ML is needed to differentiate samples from both the background and one another. In Wang *et al.*, custom electronic sensors were embedded in a microfluidic device that detected a change in current as a cell passed over electrodes within a single channel.^102^ Each sensor produced a unique 15-bit sequence that was fed into a deep CNN, which was used to predict (1) the number of cells in a single block of time; (2) the volume of each cell; (3) the velocity of the cell; and (4) the location of the cell within the device. These results were then fed to a PID controller which could change the applied pressures and subsequent velocities of cells within the device if not matching a user set-point.

High-throughput microfluidic imaging of samples is a rapid way to build a rich dataset of cell morphology and fluorescent expression, however, generally it has been a too slow process to act upon in real-time. This barrier was overcome with complex hardware and high-performance computing, creating the first image-activate cell sorting platforms within a microfluidic device.^20,24,103,104^ In this system, a custom fluorescent microscope acquired cell images in bright-field as well as two fluorescent channels at up to 18 000 frames per second. Images are reconstructed, classified using a deep CNN, and sorted with pneumatic actuators.

When microfluidics are deployed for point-of-care (POC) diagnostics, automated error detection and resolution is critical. Bhuiyan *et al.* developed a microfluidic platform for POC diagnostics that can be fully operated by a smartphone.^105^ To reduce test error from air bubbles trapped within microfluidic channels, an image-based classifier was implemented that detected bubbles within the reaction chamber and ordered an integrated pump to remove and reinsert the sample, passing by a bubble trap that removed erroneous pockets of air. By doing so, the reaction area is guaranteed to have maximal coverage, improving the reproducibility and sensitive of the device in a fully automated manner.

## 4 Outlook

ML has the potential to eliminate many of the barriers to adoption of microfluidics by non-expert users. However, there are some limitations for its use as a universal approach for microfluidic design and control. Components in microfluidics can vary significantly lab to lab, creating inconsistencies across the field that limit generalization. ML model performance is only as good as the data the models are trained on, thus large batch variability limits building high-quality cross-institutional datasets. By training on a single lab's data, the models are at a high risk for overfitting: building a dataset across a narrow distribution, models may perform well within the developer's fabrication and operational workflow but poorly in others. This effect is exacerbated by the complexity of the fluid dynamics, as performance can change significantly at different fluid properties or environmental parameters.

Extensive implementation of machine learning in microfluidics can require increased technical expertise for adopters. While tools with sophisticated GUIs are available, limitations in academic software maintenance can quickly render such tools obsolete without a user able to update the software for their own purposes. Although some additional technical skills need to be available, there is a wealth of easy-to-use ML libraries with extensive documentation, going from standard implementation (Tensorflow,^106^ PyTorch,^107^ Keras,^108^ scikit-learn^109^) to “low/no code” environments (Huggingface,^110^ Create ML,^111^ Google Cloud AutoML^112^). These packages enable microfluidic developers to quickly build or edit existing predictive models for their own applications without extensive knowledge. Here, we discuss some approaches to overcome barriers to widespread use of ML in microfluidic design and control and new application areas.

### 4.1 Transfer learning-enabled microfluidics

ML is a powerful tool to distill microfluidic expertise in an automated and reproducible manner. However, most current progress has been limited to specific components, fluid types, and operating conditions, which is not compatible with the broad application areas of microfluidics. Effective transfer of microfluidic expertise requires a streamlined process to modify ML models to small changes in the protocol, rather than having new users build their own datasets from scratch. This can be achieved with transfer learning, where a small dataset is used to retrain specific layers of a NN to adjust for difference in the dataset.^114^ Transfer learning is common outside of microfluidics; for image classification, it is standard practice to load a model pretrained on a massive dataset and transfer it to a specific application (*i.e.* using the VGG19 trained on the ImageNet dataset to specifically classify images of cats and dogs).^115,116^

**Fig. 5:**
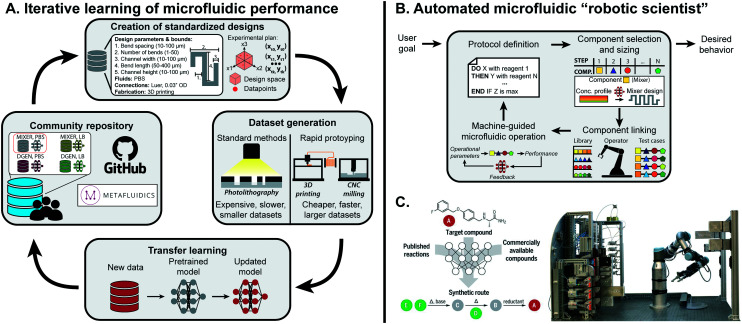
Outlook for machine learning in microfluidics. (A) To effectively learn microfluidic performance across the field, community repositories are needed consisting of standardized designs, fabrication protocols, and performance metrics together with predictive models previously trained on standardized datasets. These datasets and models can be retrieved by researchers, and adapted to new applications through transfer learning with additional smaller datasets. Sharing the updated models on community repositories completes a positive feedback cycle to continuously extend the predictive understanding of microfluidics to new components and applications. (B) Once the performance of enough components are accurately predicted, they can be integrated into an ML-guided “robot scientist” that can route together different components for fully-automated hypothesis testing and optimization. (C) Such a system has been successfully implemented in automated synthesis of organic compounds using millifluidic flow modules. From Coley *et al.*, 2019.^113^ Reprinted with permission from AAAS.

For a single microfluidic component, transfer learning could be applied to predict component performance across different fluid types, design space ranges, or substrate composition. It is possible as well for a model to be extended across components within a class of microfluidics (paper, droplet-based, *etc.*), as fundamental fluidic phenomena may be conserved.^117^ Transfer learning has been implemented for droplet generation: when changing the discrete phase used in droplet generation from DI water to LB bacterial cell media, only 36 datapoints were needed to achieve sufficient accuracy with transfer learning in contrast to the 888 originally needed to train from scratch.^13^ As the general phenomena of droplet generation is conserved between datasets, base patterns are conserved within the ML model. Building off this preliminary example, integration of rapid prototyping, community repositories, and design standardization would accelerate the feasibility and adoption of transfer learning within microfluidics (Fig. 5A).

#### 4.1.1 Rapid prototyping for data generation

Training ML models can be a data-hungry exercise and is particularly challenging in cases where data generation requires significant time or money and a pretrained model on a similar device is unavailable. In microfluidic design automation, exhaustive understanding of how a microfluidic component functions requires the fabrication of numerous devices covering the geometric design space untenable using lithographic techniques. Initial data generation with rapidly prototyped microfluidics would eliminate this barrier; the initial dataset could be made with components made from 3D printing,^118,119^ micromilling,^33,120^ or laser cutting^121^ with electronic components made from liquid metal,^122^ conductive ink,^123^ or salt water^124^ first and then the model could be refined *via* transfer learning using a much smaller dataset of devices made with photolithography and micropatterned electrodes. These datasets can further be supported by well-defined numerical models, capable of generating millions of datapoints in an efficient and inexpensive manner.

#### 4.1.2 Community repositories

Community-curated repositories of data are an essential component in the advancement of ML. Kaggle, a popular community for ML practitioners has over 100 000 public datasets applicable for most ML applications and model architectures (https://www.kaggle.com/datasets).^130^ Within the life sciences, the NIH National Center for Biotechnology Information (NCBI) curates large datasets including DNA sequences, protein composition, organism taxonomy, and more (https://www.ncbi.nlm.nih.gov/).^131^ These repositories make large datasets easily accessible, limiting the amount of data collection and preprocessing needed to train sophisticated predictive models and have been critical in the adoption of ML across disciplines.

Most journals publishing microfluidics research encourage the upload of design files and operational descriptions to improve the reproducibility of the work. However, looking up the design and operational parameters for common fluidic components used across multiple publications requires the user to search the literature and troubleshoot their device in the lab. This could be solved with a community-driven microfluidic repository that contains fully-specified device designs, flow conditions for all inputs, external controllers (pumps, circuits, *etc.*), and key performance metrics that communicate the measured output as well as any troubleshooting needed. Metafluidics is one such repository, yet it only contains designs available for user download (https://metafluidics.org).^125^ Presenting designs, operational instructions, performance metrics, and experimental data in both human and machine readable formats would allow researchers to more readily reproduce microfluidic results as well as continuously update available datasets for training of ML models.

#### 4.1.3 Design standardization

Currently microfluidic design is a manual process, building design geometries by hand using CAD tools. This, along with the bespoke nature of microfluidic development, has meant that components designed for the same purpose by different users often have small differences that may or may not affect function. Design standardization in the microfluidics community could: (1) improve the reproducibility of devices lab-to-lab, (2) make it possible to combine datasets from multiple organizations, and (3) enhance community-led microfluidic design automation.^126^

### 4.2 Fully-automated microfluidic experimentation

Thus far, efforts simplifying microfluidic design and operation with ML have mostly been on the individual component level. This approach needs to be scaled up to the system-level to achieve multi-component, fully automated lab-on-a-chip platforms. Preliminary work has implemented RL for the design of rudimentary droplet microfluidic networks, which lays the groundwork for more complex systems once individual components are fully characterized.^127^ Even with sophisticated design algorithms, combining multiple components within a single chip may not be possible: rather, utilizing a master operator capable of putting together re-configurable, modular components would significantly scale the applications of a single platform and make it more robust to individual component errors or limitations (Fig. 5B). Dictating such a flexible and high-throughput platform with ML could lead to a fully automated “robotic scientist”.^128^

Similar efforts have been shown to be effective for millifluidic flow chemistry modules (Fig. 5C). With a single component, Rizkin *et al.* optimized the catalytic productivity of metallocene-catalyzed polymerization using a machine-guided reaction chamber.^129^ The reaction was captured with an infrared camera, which was used to train a NN that optimized productivity across monomer, catalyst, and activator concentrations as well as temperature. This approach was scaled up by Coley *et al.*, which combined multiple millifluidic components, ML-based protocol planning, and a robotic operator to design, configure, and execute organic chemical synthesis.^113^ Existing components could be switched out with more complex fluidic systems, such as droplet microfluidics, to shift the paradigm of testing in life and chemical sciences.

## Conflicts of interest

There are no conflicts to declare.

## Supplementary Material
